# Indications of Th1 and Th17 responses in cerebrospinal fluid from patients with Lyme neuroborreliosis: a large retrospective study

**DOI:** 10.1186/1742-2094-8-36

**Published:** 2011-04-20

**Authors:** Anna J Henningsson, Ivar Tjernberg, Bo-Eric Malmvall, Pia Forsberg, Jan Ernerudh

**Affiliations:** 1 Department of Infectious Diseases, Ryhov County Hospital, Jönköping, Sweden; 2 Department of Clinical Microbiology, Ryhov County Hospital, Jönköping, Sweden; 3 Department of Clinical Chemistry, Kalmar County Hospital, Kalmar, Sweden; 4 Futurum Academy of Health Care, Ryhov County Hospital, Jönköping, Sweden; 5 Division of Infectious Medicine, Department of Clinical and Experimental Medicine, Linköping University, Sweden; 6 Division of Clinical Immunology, Department of Clinical and Experimental Medicine, Linköping University, Sweden

## Abstract

**Background:**

Previous studies indicate that successful resolution of Lyme neuroborreliosis (NB) is associated with a strong T helper (Th) 1-type cytokine response in the cerebrospinal fluid (CSF) followed by a down-regulating Th2 response, whereas the role of the recently discovered Th17 cytokine response is unknown.

**Methods:**

To investigate the relative contribution of different Th associated cytokine/chemokine responses, we used a multiple bead array to measure the levels of CXCL10 (Th1 marker), CCL22 (Th2 marker), IL-17 (Th17 marker) and CXCL8 (general inflammation marker), in serum and in CSF from untreated patients with confirmed NB (n = 133), and non-NB patients (n = 96), and related the findings to clinical data. Samples from patients with possible early NB (n = 15) and possible late NB (n = 19) were also analysed, as well as samples from an additional control group with orthopaedic patients (n = 17), where CSF was obtained at spinal anaesthesia.

**Results:**

The most prominent differences across groups were found in the CSF. IL-17 was elevated in CSF in 49% of the patients with confirmed NB, but was not detectable in the other groups. Patients with confirmed NB and possible early NB had significantly higher CSF levels of CXCL10, CCL22 and CXCL8 compared to both the non-NB group and the control group (p < 0.0001 for all comparisons). Patients in the early NB group, showing a short duration of symptoms, had lower CCL22 levels in CSF than did the confirmed NB group (p < 0.0001). Furthermore, patients within the confirmed NB group showing a duration of symptoms <2 weeks, tended to have lower CCL22 levels in CSF than did those with longer symptom duration (p = 0.023). Cytokine/chemokine levels were not correlated with clinical parameters or to levels of anti-*Borrelia*-antibodies.

**Conclusion:**

Our results support the notion that early NB is dominated by a Th1-type response, eventually accompanied by a Th2 response. Interestingly, IL-17 was increased exclusively in CSF from patients with confirmed NB, suggesting a hitherto unknown role for Th17 in NB. However, for conclusive evidence, future prospective studies are needed.

## Background

Neuroborreliosis (NB) is the most common manifestation of disseminated borreliosis in Europe [1,2]. Most patients recover after antibiotic treatment, while some experience persisting symptoms despite adequate therapy [3-8]. The pathogenic mechanisms behind the variable outcome are not fully understood. Previous studies have indicated that a good prognosis in NB seems to be associated with a strong T helper (Th) 1-type immune response in the cerebrospinal fluid (CSF) early in the infection [9-15], followed by a Th2-type response, capable of suppressing the Th1-type inflammation. If this switching is delayed, there is a risk of tissue damage and persisting symptoms [16-19].

The Th1/Th2 concept has recently been extended to include a population called Th17, based on their secretion of interleukin (IL)-17 [20]. Th17 cells are thought to play a key role in the induction and development of tissue injury in some autoimmune diseases, although so far mainly shown in experimental models [21,22]. Recent studies have also demonstrated induction of IL-17 preferentially in infections with extra-cellular bacteria and fungi [23]. It has been suggested that Th17 cells and their associated cytokines are involved in the pathogenesis of Lyme arthritis [24-26], whereas data on Th17 involvement in NB is lacking.

Chemokines are small chemotactic cytokines that are induced during an immune response to promote migration of immune cells to the site of infection [27]. Chemokines have a crucial role in establishing the Th1/Th2 balance and they are used as markers for Th1/Th2 immunity. The chemokine CXCL10 (IFN-γ inducible protein 10, IP-10) is secreted by several cell types, *e.g*. monocytes, endothelial cells and fibroblasts [28] in response to IFN-γ, and plays an important role in attracting T cells into sites of Th1-type inflammation [29]. Previous studies have indicated the presence of CXCL10 in CSF from NB patients [30] as well as in skin samples from patients with dermatoborreliosis [31]. The Th2-associated chemokine CCL22 (macrophage-derived chemokine, MDC) is secreted by dendritic cells and macrophages [32], and is a chemoattractant for monocytes, immature dendritic cells and natural killer cells [33]. CXCL8 (IL-8) is secreted by several cell types, e.g. macrophages, dendritic cells and endothelial cells [34,35]. Its primary function is to recruit neutrophil granulocytes early in the inflammation process [36], and CXCL8 can therefore be regarded as a general and early marker of inflammation. Furthermore, CXCL8 is probably the most important neutrophil-attracting factor induced by IL-17 [37].

The aim of this study was to assess Th associated cytokine/chemokine profiles in serum and in CSF in NB patients in the framework of a large retrospective study. The relative contribution of Th1-, Th2-, and Th17-like responses were estimated by the levels of CXCL10, CCL22 and IL-17A (here referred to as IL-17), respectively. CXCL8 was analyzed as a general marker of inflammation. In addition, we also wanted to relate the cytokine/chemokine levels to age, sex, clinical presentation and course of the disease.

## Methods

### Patients

Serum and CSF samples of 263 patients were investigated for suspected NB during 2003 through 2005 at the hospitals of Kalmar (n = 165) and Jönköping (n = 98). The domiciles of the patients were distributed all over the Counties of Kalmar and Jönköping, respectively. None of the patients had, in conjunction with the current study, received antibiotic treatment for NB prior to the lumbar puncture (LP). Elevated anti-*Borrelia *antibody index (AI) or increased levels of *Borrelia*-specific IgG or IgM antibodies in CSF were found in 152 patients, whereas 111 patients had no detectable *Borrelia*-specific antibodies in CSF. In the entire study group (n = 263), there were 155 men (59%) and 108 women (41%). The median age in the whole group was 41 years, range 2-87 years. There were 95 (36%) patients aged 15 years or less, and 168 (64%) patients were over 15 years of age. The patients were divided into four groups based on the CSF findings (Table 1). Patients in group 1 had both detectable *Borrelia*-specific antibodies in CSF and pleocytosis. Group 2 had *Borrelia*-specific antibodies in CSF, but no pleocytosis. Group 3 consisted of 15 children with symptoms strongly indicative of NB, *i.e*. subacute meningitis and/or facial palsy. They had pleocytosis but no detectable *Borrelia*-specific antibodies in CSF. Group 4 consisted of patients without intrathecal anti-*Borrelia *antibodies and no pleocytosis, thereby constituting a non-NB group. They had been investigated for symptoms suggestive of NB, such as facial palsy, headache, muscle and joint pain, vertigo or fatigue. In addition to these four groups we used a control group (group 5) where CSF was not obtained due to suspicion of NB; 17 patients undergoing elective orthopaedic surgery, where CSF was collected prior to spinal anaesthesia. The reference material was collected at the University Hospital of Linköping from patients that had not experienced NB and they were negative for anti-*Borrelia *antibodies in serum and in CSF. Further characteristics of the patient groups are presented in Table 1.

**Table 1:** Characteristics of the different study groups.

	Group 1 Confirmed NB *n = 133*	Group 2 Possible late NB *n = 19*	Group 3 Possible early NB *n = 15*	Group 4 Non-NB *n = 96*	Group 5 Control patients *n = 17*
*Borrelia*-specific AI or *Borrelia*-specific anti- bodies in CSF	+	+	-	-	-
CSF pleocytosis	+	-	+	-	-
CSF-albumin S-albumin median, (range)	16*** (3.4-76)	5.5 (2.4-21)	3.5 (0-12)	4.3 (0-22)	n.d.
*Borrelia*-specific IgG/IgM antibodies detected in serum; *n*, (%)	115*** (87)	15* (79)	15*** (100)	42 (44)	0*** (0)
Men *n*, (%)	80 (60)	14 (74)	9 (60)	52 (54)	9 (53)
Women *n*, (%)	53 (40)	5 (26)	6 (40)	44 (46)	8 (47)
Median age years, (range)	35** (3-87)	52 (18-76)	7*** (2-13)	44 (2-83)	66* (50-72)
Median duration of symptoms before LP; weeks, (range)	2.4 (0-32)	4.0 (0.1-77)	0.7 (0.1-4.0)	3.5 (0-730)	-
Head/neck pain *n*, (%)	77 (58)	11 (58)	11 (73)	43 (45)	-
Cranial nerve palsy *n*, (%)	70 (53)	4 (21)	11 (73)	13 (14)	-
Radiculitis *n*, (%)	53 (40)	7 (37)	0 (0)	3 (3)	-
Other symptom *n*, (%)	9 (7)	2 (11)	0 (0)	39 (41)	-

NB: neuroborreliosis.

*n*: number of patients.

AI: antibody index.

CSF:cerebrospinal fluid.

Pleocytosis:>5 mononuclear cells/μL CSF.

S: serum.

LP: lumbar puncture.

n.d: not done.

Patients could have one or more of the symptoms head/neck pain, cranial nerve palsy and radiculitis. Patients with none of the above symptoms were classified as "other symptom" (*e.g*. vertigo, muscle and joint pain, parestesias, fatigue, dementia or concentration difficulties).

* significant differences compared to the non-NB group (group 4). * p < 0.01, **p < 0.001, ***p < 0.0001.

### Serum and CSF samples

The Clinical Laboratory of Microbiology (CLM) in Kalmar used an ELISA kit measuring intrathecal production of *Borrelia*-specific antibodies, *i.e*. AI, (IDEIA Lyme Neuroborreliosis, K6028, Dako Cytomation, UK). Results were interpreted as negative or positive according to the manufacturer's instructions. Serum samples from patients in Kalmar County were investigated with Immunetics Quick ELISA Borrelia C6 Assay kit, Immunetics, Cambridge, MA, USA.

At the CLM in Jönköping the Lyme Borreliosis ELISA kit 2^nd ^Generation (Dako Cytomation, A/S, Glostrup, Denmark) was used for measuring *Borrelia*-specific antibodies in serum and in CSF. Results were interpreted as positive or negative according to a cut-off adjusted to a local sample collection used when validating the method.

The control samples (group 5) were analysed for *Borrelia*-specific antibodies by ELISA kit measuring serum and intrathecal production of antibodies (IDEIA Lyme Neuroborreliosis, K6028, Dako Cytomation, UK).

All samples had been stored at -20°C and thawed once before the current analyses. The cytokine/chemokine and albumin analyses were performed in 2009 using Luminex technology (see below) and rate nephelometry (Beckman Coulter Immage 800), respectively.

### Cytokine/chemokine measurements

Concentrations were measured by Luminex multiple bead technology (Milliplex Human Cytokine/Chemokine Kit, Millipore Corporation) according to the instructions provided by the manufacturer. The lower detection limits were as follows; CXCL8: 1.6 pg/mL, IL-17: 1.6 pg/mL, CXCL10: 16 pg/mL, CCL22: 80 pg/mL in serum, 20 pg/mL in CSF. Values under the detection limit were given half the value of the lowest point of the standard curve. The inter-assay coefficient of variation (CV) was 9.3-11.6%, and the intra-assay CV was 4.5-7.1% according to the manufacturer. In the following, CXCL8, CXCL10 and CCL22 are collectively referred to as chemokines; IL-17 is referred to as cytokine, whereas all (CXCL8, CXCL10, CCL22 and IL-17) are referred to as cytokine/chemokines.

### Data handling and statistics

Statistical analyses were performed using SPSS for Windows, version 15.0. Since most of the variables had skewed distributions, non-parametric tests were used. Kruskal-Wallis ANOVA was performed to compare multiple study groups, and Mann-Whitney U test was applied as a post-hoc test. For ordered categorical variables, the Chi^2 ^test was used. Correlations between parameters were calculated using Spearman correlation analysis (rho values are given). P-values < 0.01 were considered to be significant, this p-value was chosen to avoid mass-significance problems. By analogy to the IgG-index used for calculations of intrathecal IgG production [38], we calculated a "chemokine intrathecal production index" using the formula (CSF-chemokine/S-chemokine)/(CSF-albumin/S-albumin). The CCL22 index was multiplied by 100 due to the much higher concentration in serum. IL-17 was not detectable in CSF in three of the four patient groups, and thus no data are given for the IL-17 intrathecal production index. Albumin values were not available in all samples from the control group (group 5).

### Ethics

The study was approved by the Regional Ethical Review Board in Linköping, Sweden.

## Results

There were no differences in cytokine/chemokine levels in serum or in CSF between men and women. When comparing the patients under 15 years of age to patients over 15 in the entire study population (groups 1-5), the children had significantly higher CSF levels of CXCL8 (p < 0.0001), CXCL10 (p < 0.0001), CCL22 (p = 0.0001) and IL-17 (p = 0.001). In serum, there were no differences in cytokine/chemokine levels between younger or older patients, except for CCL22 that was higher in the patients under 15 years of age (p < 0.0001).

**Figure 1:**
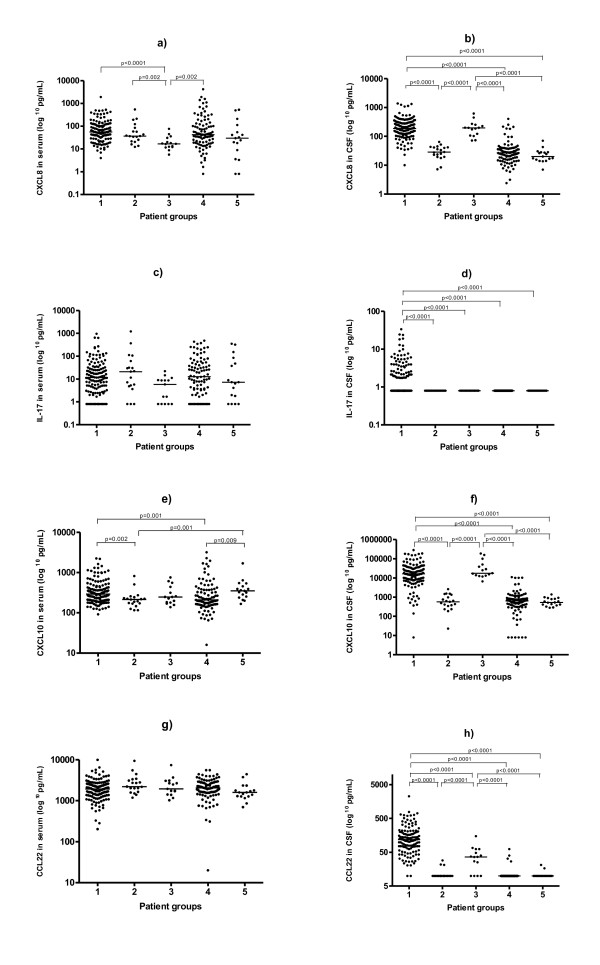
**Cytokine/chemokine levels (pg/mL) in serum and in cerebrospinal fluid (CSF)**. Group 1: Confirmed neuroborreliosis (NB), patients with elevated *Borrelia*-specific antibody index or *Borrelia*-specific antibodies in CSF and pleocytosis. Group 2: Possible late NB, patients with elevated *Borrelia*-specific antibody index or *Borrelia*-specific antibodies in CSF but no pleocytosis. Group 3: Possible early NB, children with CSF pleocytosis but no detectable *Borrelia*-specific antibodies in CSF. Group 4: Non-NB, patients without pleocytosis and no detectable *Borrelia*-specific antibodies in CSF. Group 5: Control group, CSF was obtained at spinal anaesthesia from patients undergoing elective orthopaedic surgery. Bars represent the median cytokine/chemokine level in each group.

The cytokine/chemokine data from the aspect of the different study groups are presented in Figure 1. In serum, there were few significant differences across groups; the CXCL8 levels were decreased in group 3, children with possible early NB (Figure 1a), whereas the CXCL10 levels were increased in group 1, confirmed NB and in group 5, the control group (Figure 1e). There were no significant differences across groups regarding IL-17 or CCL22 levels in serum.

In contrast to serum, CSF showed several significant differences across the groups. Groups 1 and 3, *i.e*. confirmed NB and possible early NB, showed increased levels of CXCL8 (Figure 1b) and CXCL10 (Figure 1f) as compared to the other groups. For CCL22, the highest levels were found in group 1, but CCL22 was also elevated in group 3. Thus, regarding the kinetics of CCL22, moderate levels of CCL22 were associated with short symptom duration since group 3, with the shortest symptom duration (p < 0.0001 when compared to group 1), showed lower CCL22 levels than group 1. In addition, patients within the confirmed NB group showing a duration of symptoms <2 weeks, also tended to have lower CCL22 CSF levels than did those with a longer symptom duration (p = 0.023). Notably, IL-17 levels were significantly higher in group 1, confirmed NB, while it was not detectable in CSF of the other groups (Figure 1d).

All the cytokine/chemokine levels in CSF correlated with CSF pleocytosis when looking at the entire study population, *i.e*. CXCL8: rho = 0.71, IL-17: rho = 0.54, CXCL10: rho = 0.71, CCL22: rho = 0.83 (all p values <0.0001, data not shown in figures). Furthermore, cytokine/chemokine levels in CSF did not correlate with the corresponding serum levels. The chemokine intrathecal production indices for CXCL8, CXCL10 and CCL22 were increased in groups 1 and 3 (Table 2), *i.e*. confirmed NB and possible early NB.

**Table 2:** CSF-albumin/S-albumin ratios and chemokine intrathecal production indices for the different patient groups^¤^.

	Group 1 Confirmed NB *n = 133*	Group 2 Possible late NB *n = 19*	Group 3 Possible early NB *n = 15*	Group 4 Non-NB *n = 96*
CXCL8 index median, (range)	0.23* (0.01-2.3)	0.11 (0.01-0.5)	4.56*** (0.19-8.63)	0.16 (0-7.40)
CXCL10 index median, (range)	3.13*** (0-76.7)	0.42 (0.02-1.08)	14.4*** (5.21-96.4)	0.59 (0-10.9)
CCL22 index median, (range)	0.30*** (0-7.00)	0.07 (0-0.20)	0.20*** (0-2.00)	0.10 (0-7.00)

Chemokine index: (CSF-chemokine/S-chemokine)/(CSF-albumin/S-albumin).

NB: neuroborreliosis.

*n*: number of patients.

CSF: cerebrospinal fluid.

S: serum.

¤: Indices could not be calculated for group 5, since CSF-albumin/S-albumin ratios were not available for all.

The CCL22 index was multiplied by 100 due to much higher concentrations in serum.

The IL-17 index was not calculated since IL-17 was not detectable in CSF in groups 2, 3 and 4.

* significant differences compared to the non-NB group (group 4). * p < 0.01, ** p < 0.001, *** p < 0.0001.

Group 1, the confirmed NB group, showed significantly higher CSF levels of all cytokine/chemokines compared with groups 4, the non-NB group, and 5, the control group (Figure 1). However, regarding clinical parameters, there were no correlations within group 1 between cytokine/chemokine levels in CSF and either *i*) age, *ii*) duration of symptoms prior to diagnosis, *iii*) degree of pleocytosis in CSF, *iv*) intrathecal *Borrelia*-specific antibodies, or *v*) duration of symptoms after treatment (data not shown). Group 1 also showed increased CSF levels of IL-17, being detected (*i.e*. >0.80 pg/mL) in 65 patients (49%), whereas IL-17 was not detected in CSF of the other groups. However, there were no significant differences between patients with elevated IL-17 levels in CSF and the remaining group 1 patients regarding age, sex, duration of symptoms prior to diagnosis or duration of symptoms after treatment.

Patients with elevated IL-17 in CSF had more pronounced pleocytosis (p = 0.004) compared to the patients in group 1 with undetectable levels of IL-17 in CSF. There was no correlation within group 1 between serum and CSF levels of IL-17 (rho = 0.09, p = 0.316), or between the CSF-albumin/S-albumin ratio and IL-17 in CSF; (rho = 0.18, p = 0.045). Further, there was no significant difference in CSF-albumin/S-albumin ratio when comparing patients with elevated IL-17 in CSF to those with low levels (p = 0.172) (Table 3). There were no differences in clinical presentation between patients with high or low CSF-IL-17, except for fatigue, being more common in the group with high IL-17 levels (Table 3).

**Table 3:** Clinical features of patients with confirmed neuroborreliosis, stratified into two groups depending on the IL-17 level in cerebrospinal fluid.

	IL-17 in CSF <0.80 pg/mL *n = 68*	IL-17 in CSF >0.80 pg/mL *n = 65*
Pleocytosis median number of cells/μL, (range)	138 (8-384)	184* (10-1650)
CSF-albumin S-albumin median, (range)	14.3 (3.4-59.6)	18.1 (3.8-76.0)
Median age years, (range)	48 (3-87)	14 (3-77)
Median duration of symptoms before LP weeks, (range)	2.0 (0.1-25.0)	3.0 (0-32.0)
Cranial nerve palsy *n*, (%)	37 (54.4)	33 (50.8)
Muscle and joint pain *n*, (%)	32 (47.1)	27 (41.5)
Radiculitis *n*, (%)	30 (44.1)	23 (35.4)
Neck pain *n*, (%)	22 (32.4)	25 (38.5)
Headache *n*, (%)	26 (28.2)	29 (44.6)
Fatigue *n*, (%)	17 (25.0)	31* (47.7)
Parestesias *n*, (%)	12 (17.6)	11 (16.9)
Fever >38°C *n*, (%)	10 (14.7)	16 (24.6)
Vertigo *n*, (%)	4 (5.9)	6 (9.2)
Concentration difficulties *n*, (%)	2 (2.9)	2 (3.1)

CSF: cerebrospinal fluid.

LP: lumbar puncture.

S: serum.

*n*: number of patients.

°C: degrees Celsius.

* significant differences between the two groups. * p < 0.01, ** p < 0.001, *** p < 0.0001.

Patients could have one or several symptoms.

Group 2, possible late NB, did not differ from the non-NB group or the control group in their serum or CSF levels regarding cytokine/chemokine levels or intrathecal indices, except for higher serum levels of CXCL10 in the control group (Figure 1e).

**Figure 2:**
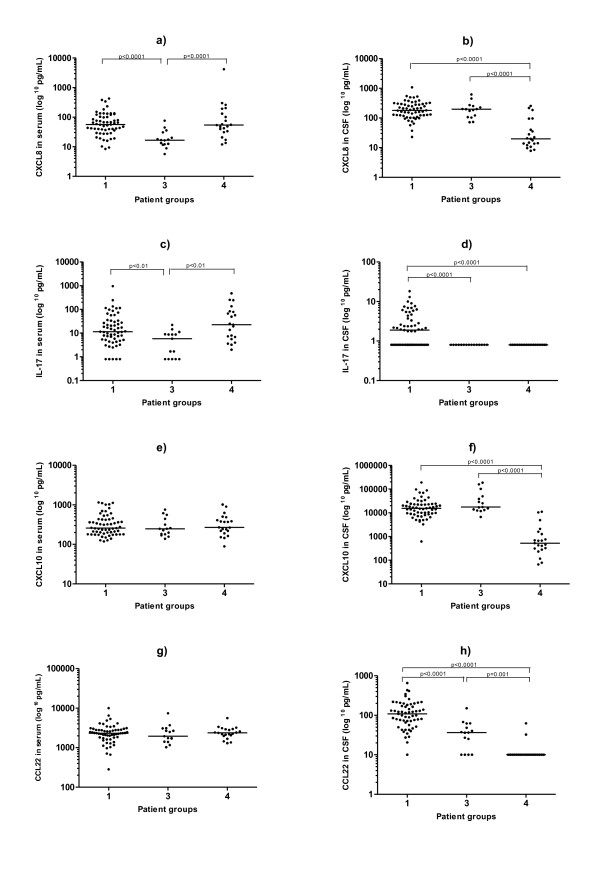
**Cytokine/chemokine levels (pg/mL) in serum and in cerebrospinal fluid (CSF) in patients <15 years of age**. Group 1: Confirmed neuroborreliosis (NB), patients with elevated *Borrelia*-specific antibody index or *Borrelia*-specific antibodies in CSF and pleocytosis. Group 3: Possible early NB, patients with CSF pleocytosis but no detectable *Borrelia*-specific antibodies in CSF. Group 4: Non-NB, patients without pleocytosis and no detectable *Borrelia*-specific antibodies in CSF. (There were no children in groups 2 and 5). Bars represent the median cytokine/chemokine level in each group.

Since group 3 only consisted of children, we also made comparisons between group 3 and the children in groups 1 and 4, respectively (there were no children in groups 2 and 5). The same differences between the groups were obtained as when analysing the entire study population (Table 4 and Figure 2).

**Table 4:** Characteristics of the children under 15 years of age^¤^

	Group 1 Confirmed NB *n = 59*	Group 3 Possible early NB *n = 15*	Group 4 Non-NB *n = 21*
*Borrelia*-specific AI or *Borrelia*-specific anti- bodies in CSF	+	-	-
CSF pleocytosis	+	+	-
CSF-albumin S-albumin median, (range)	11*** (3.4-31)	3.5 (0-12)	3.1 (0-22)
Men *n*, (%)	33 (60)	9 (60)	8 (38)
Women *n*, (%)	26 (40)	6 (40)	13 (62)
Median age years, (range)	9 (3-14)	7 (2-13)	9 (2-13)
Median duration of symptoms before LP; weeks, (range)	2.0 (0-8.0)	0.7 (0.1-4.0)	1.7 (0-13)

¤ There were no children in groups 2 and 5.

NB: neuroborreliosis.

*n*: number of patients.

AI: antibody index.

CSF:cerebrospinal fluid.

Pleocytosis:>5 mononuclear cells/μL CSF.

S: serum.

LP: lumbar puncture.

* significant differences compared with the non-NB group. * p < 0.01, ** p < 0.001, *** p < 0.0001.

## Discussion

In this study we aimed at elucidating the balance of cytokine/chemokines related to different Th cell populations including the recently described Th17 population. For this purpose, a large material of collected serum and CSF samples from patients with clinically suspected NB was used. The age and sex distribution were in accordance with a previous study from the areas around Kalmar and Jönköping [1], supporting that the study population is representative for NB patients. The patients were stratified according to the primary findings in the CSF. One large group (group 1) was regarded as "confirmed NB" since these patients showed both CSF pleocytosis and presence of intrathecal anti-*Borrelia *antibodies [39]. The patients in group 2 had *Borrelia*-specific antibodies in CSF, but no pleocytosis. They had slightly longer duration of symptoms prior to diagnosis compared to group 1, so in their case it is possible that a previous pleocytosis had resolved by the time of the LP, probably reflecting the natural course of NB in most cases, even without treatment. Taking this into consideration, group 2 could be regarded as patients with "possible late NB", although a number of cases could well have been "status post" NB, and thus, we could not draw any firm conclusions from this group. The patients in group 3 were all children showing CSF pleocytosis but no detectable *Borrelia*-specific antibodies in CSF. They had a significantly shorter duration of symptoms prior to the LP compared to group 1. Their clinical symptoms were strongly indicative of NB; in all cases subacute meningitis and/or facial palsy, a symptom that in Scandinavia is commonly caused by *Borrelia *in paediatric patients [40,41]. These patients were therefore regarded as "possible early NB". In further support of this notion, we previously demonstrated that a similar group of children with "possible NB" revealed *Borrelia*-specific T-cell responses to the same extent as did children with "confirmed NB" [42]. Another large group (group 4, the non-NB group) could be regarded as a kind of control group, since these patients had no signs of CNS inflammation and no intrathecal *Borrelia*-specific antibodies. Group 4 consisted of patients investigated for symptoms suggestive of NB, but without serologic findings of current NB. A significantly larger proportion of the patients in groups 1, 2 and 3 had detectable *Borrelia*-specific antibodies in serum compared to the patients in group 4. However, the presence or absence of anti-*Borrelia *antibodies in serum should be interpreted with caution, since the study is carried out in a highly endemic area where patients could have been exposed to *Borrelia *previously. Further, NB patients may develop anti-*Borrelia *antibodies in CSF before the antibodies are detectable in serum [43]. Group 5 consisted of patients without a medical history of NB and no current symptoms suggestive of NB. They did not have CSF pleocytosis or detectable *Borrelia*-specific antibodies in serum or in CSF, and were therefore included as an additional and independent control group.

The most prominent differences across groups were found in CSF. CXCL8 and CXCL10 were significantly elevated in group 1 (confirmed NB) and in group 3 (possible early NB). CCL22 in CSF was particularly elevated in group 1, and moderately elevated in group 3. Here, the time aspect is probably of importance for the chemokine profile. Early in the inflammation process, a Th1-type immune response dominates (as in group 3, possible early NB), but is then counter-balanced by a Th2 response (as in group 1, confirmed NB). This pattern is also found within group 1, *i.e*. patients with duration of symptoms <2 weeks also tended to have lower CCL22 CSF levels than those with longer symptom duration. This scenario corroborates previous observations and is in line with the hypothesis that Th2 controls the primary Th1 response, the latter being crucial in bacteria elimination but also potentially involved in tissue damage [12-19].

Remarkably, half of the patients in group 1 had elevated levels of IL-17 in CSF, whereas IL-17 was not detectable in any of the other patient groups. The role of the increase in IL-17 is still puzzling since there were no significant differences between those with high levels of IL-17 in CSF and those with low levels regarding sex, age, duration of symptoms prior to diagnosis, or duration of symptoms after treatment. The significant findings distinguishing patients with high levels of CSF-IL-17 from those with low levels, were a more pronounced pleocytosis, slightly more elevated CXCL10 in CSF and a higher frequency of fatigue. These findings are hard to interpret and need to be confirmed. The association with pleocytosis suggests IL-17 to be associated with ongoing inflammation, although the absence of IL-17 in children with pleocytosis but no anti-*Borrelia*-antibodies in CSF (group 3) is then puzzling. However, it could be speculated that children show defective or delayed Th17 responses. The presence of IL-17 in CSF of NB patients has only recently been reported in another cohort of NB patients on the Islands of Åland [44]. Since IL-17 has proven important in the context of extra-cellular bacteria, and in the induction of immune-mediated tissue injury [45], further studies on IL-17 and its role in the pathogenesis and clinical out-come of NB are required.

CXCL8 is one important effector mechanism associated with IL-17, which is in line with the high CXCL8 levels in group 1. However, also the patients in group 3 (possible early NB) showed high levels of CXCL8 in CSF, but no detectable IL-17, which suggests that CXCL8 might be induced by other pathways independent of IL-17.

We found several evidence supporting an intrathecal origin of the induced cytokine/chemokines; no correlations between serum and CSF levels, the levels being of the same magnitude (or higher) in CSF compared to serum for CXCL8, CXCL10 and IL-17, and increased chemokine intrathecal production indices for CXCL8, CXCL10 and CCL22 in groups 1 and 3. For IL-17, an intrathecal production index could not be reliably calculated due to the absence of IL-17 in CSF of several groups. Yet, it seems that IL-17 is produced intrathecally since neither the serum levels of IL-17 nor the CSF-albumin/S-albumin ratios correlated with the CSF levels of IL-17.

As to findings in serum, the patients with confirmed NB showed significantly higher levels of CXCL10 as compared to groups 2 and 4, possibly reflecting that the strong Th1 response in the CNS also is associated with a systemic response. However, the orthopaedic control patients also displayed elevated CXCL10 levels in serum. It could be speculated that this finding might be related to age [46]. Surprisingly, children with possible early NB (group 3) showed significantly lower levels of CXCL8 in serum, and this finding persisted in the comparison across groups 1, 3 and 4 when all adult patients were excluded. Otherwise, no significant differences were found across groups for cytokine/chemokine levels in serum, confirming that the inflammatory process is mainly restricted to the CNS compartment [13].

We also attempted to find correlations between cytokine/chemokine levels and clinical parameters including the course of disease. However, no such correlations were found, which implies that cytokine/chemokine levels do not seem to be useful clinical predictors of the disease course. However, we can not exclude that such correlations would have been revealed if a standardized follow-up protocol had been applied in a prospective manner.

One limitation to this study is the difficulty to find an appropriate control group. The use of completely healthy control patients with no symptoms and no other disorders would of course be desirable, but it is generally very difficult to obtain CSF samples from healthy volunteers. Importantly, we included a control group (group 5) where CSF was not obtained due to suspicion of NB. We also believe that our non-NB group (group 4) is quite useful, since the patients have neither signs of ongoing *Borrelia *infection, nor any signs of CNS inflammation, and it is our opinion that the inflammation in NB is mainly restricted to the CNS compartment. Furthermore, there were no significant differences found between the non-NB group and the control group (group 5), except for higher serum levels of CXCL10 in the control group as discussed above. Another possible limitation to this study is the difference in age between patients in group 3 (children with possible early NB) and patients in the other groups. However, when comparing group 3 to the children in groups 1 and 4, we obtained the same results as when including the adults in the analysis.

The samples had been stored at -20°C and thawed once before. However, chemokines are fairly robust, and concentrations were measurable using the methods described here. Furthermore, all samples had been handled and stored under the same conditions. It is also worth noting that we here measured the concentrations of circulating cytokine/chemokines, and the results could be different at a cellular level. However, CXCL10 and CCL22 seem to roughly reflect previous data on Th1 and Th2 [9-15].

## Conclusions

Our results support the notion that a Th1-type immune response dominates in the CNS in early NB, and is then followed by a Th2-type immune response. We suggest that chemokines are suitable and feasible markers for measuring of Th1/Th2 responses. We also show that IL-17 levels are increased in CSF in a substantial proportion of NB cases, suggesting a role for Th17 in NB. However, the precise role of Th17 in NB pathogenesis and clinical course remains to be evaluated.

## Competing interests

The authors declare that they have no competing interests.

## Consent

The study was approved by the Regional Ethical Review Board in Linköping, Sweden. A copy of the written approval is available for review by the Editor-in-Chief of this journal. The patients had given a general consent to their samples being stored in a biobank and used for research purposes.

## Authors' contributions

AJH participated in the design of the study, performed the statistical analysis and drafted the manuscript. IT, BEM and PF participated in the analysis and interpretation of data and helped to draft the manuscript. JE conceived of the study, participated in its design and coordination and helped to draft the manuscript. All authors have read and approved of the final manuscript.
